# Statistical learning for vocal sequence acquisition in a songbird

**DOI:** 10.1038/s41598-020-58983-8

**Published:** 2020-02-10

**Authors:** Logan S. James, Herie Sun, Kazuhiro Wada, Jon T. Sakata

**Affiliations:** 1 Department of Biology, McGill University, Montreal, Canada; 2 Centre for Research in Brain, Language, and Music, McGill University, Montreal, Canada; 3 Faculty of Science, Hokkaido University, Sapporo, Japan; 4 Present Address: Department of Integrative Biology, University of Texas at Austin, Austin, USA; 5 Present Address: Joan & Sanford I. Weill Medical College of Cornell University, Cornell University, New York, USA

**Keywords:** Learning and memory, Sensory processing, Animal behaviour

## Abstract

Birdsong is a learned communicative behavior that consists of discrete acoustic elements (“syllables”) that are sequenced in a controlled manner. While the learning of the acoustic structure of syllables has been extensively studied, relatively little is known about sequence learning in songbirds. Statistical learning could contribute to the acquisition of vocal sequences, and we investigated the nature and extent of sequence learning at various levels of song organization in the Bengalese finch, *Lonchura striata* var. *domestica*. We found that, under semi-natural conditions, pupils (sons) significantly reproduced the sequence statistics of their tutor’s (father’s) songs at multiple levels of organization (e.g., syllable repertoire, prevalence, and transitions). For example, the probability of syllable transitions at “branch points” (relatively complex sequences that are followed by multiple types of transitions) were significantly correlated between the songs of tutors and pupils. We confirmed the contribution of learning to sequence similarities between fathers and sons by experimentally tutoring juvenile Bengalese finches with the songs of unrelated tutors. We also discovered that the extent and fidelity of sequence similarities between tutors and pupils were significantly predicted by the prevalence of sequences in the tutor’s song and that distinct types of sequence modifications (e.g., syllable additions or deletions) followed distinct patterns. Taken together, these data provide compelling support for the role of statistical learning in vocal production learning and identify factors that could modulate the extent of vocal sequence learning.

## Introduction

Behaviors are comprised of motor gestures that are sequenced in precise ways, and the manner in which gestures are sequenced can provide information about an individual (e.g., species, individual identity, motivation)^1–5^ as well as about environmental context^6,7^. For many important behaviors, including social and communicative behaviors, the structure and sequencing of individual motor gestures can be learned. For example, speech and birdsong are communicative behaviors that consists of precisely controlled sequences of learned vocal gestures^8–10^. Despite the importance of sequencing in communication, relatively little is known about the nature of sequence learning in vocal production learning.

Statistical learning has been proposed as a mechanism for learning complex behavioral sequences, including speech and language acquisition^11–14^. In particular, young infants demonstrate a remarkable ability to distinguish auditory sequences based on the transition probabilities between various acoustic elements, and this learning is thought to scaffold subsequent word and sentence learning^15,16^. The term “statistical learning” is commonly used to describe a learning mechanism that uses statistical properties of stimuli as cues for segregating or classifying elements, but regardless of the perceptual or behavioral outcome, this process involves the ability of the nervous system to extract sequence patterns. This ability to track and remember sequences of stimuli has also been revealed in a variety of other species, including non-human primates, rodents and birds^14,17–22^. Of particular relevance is that songbirds like the Bengalese finch have been found to use statistical (or prosodic) cues to learn phrases or “chunks” of syllables^23^. However, little is known about whether learning the statistics of sensory stimuli translates into changes in vocal production in non-human animals.

Songbirds are a valuable model system to study statistical learning in the context of vocal production learning^24,25^. Songbirds learn their communication signals (“songs”) during development, and their songs consist of acoustic elements (“syllables”) that are arranged in various types of sequences, ranging from stereotyped and linear sequences (e.g., white-crowned sparrows, zebra finches) to more complex and variable sequences (e.g., nightingales, Bengalese finches)^26^. While much is known about the factors that influence how songbirds acquire the acoustic structure of song syllables, relatively few studies examine how songbirds learn to sequence these syllables. Some studies using experimental tutoring have confirmed the developmental acquisition of vocal sequencing^27–31^, but most of such studies have been conducted on species that produce stereotyped, relatively simple sequences of syllables (e.g., white-crowned sparrows and zebra finches)^26^. Understanding sequence learning in songbird species that produce more complex and variable sequencing is important not only for comparative reasons but also because such species produce syntactic structures that more closely resemble the complexity of syntax in human language^14^.

**Figure 1:**
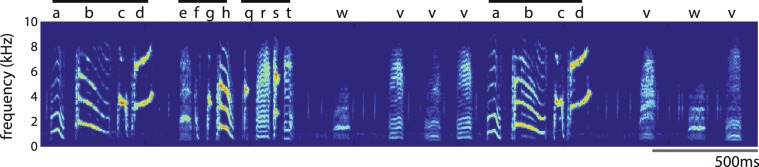
A spectrogram (time on x-axis, frequency on y-axis, color indicates intensity) of a rendition of Bengalese finch song. Letters above individual syllable types indicate arbitrary labels used for analysis. Black bars indicate the motifs “abcd”, “efgh” and “qrst”. This rendition of song provides examples of branch point transitions in Bengalese finch song, as the bird transitions from the branch point sequence (motif) “abcd” to either “e” or “v”.

The Bengalese finch (*Lonchura striata* var. *domestica*) offers an excellent opportunity to study the acquisition of relatively complex vocal sequences^32^. The songs of individual Bengalese finches consist of a discrete, learned repertoire of syllables that are arranged both in highly stereotyped sequences (“motifs” or “chunks”) and in sequences that vary from rendition to rendition (“branch points”; Fig. 1)^26,33–40^. Previous studies have provided some insight into vocal sequence learning in Bengalese finches by comparing songs between tutors and pupils^41–43^; for example, Yamashita *et al*. (2008) analyzed the distribution of three-syllable sequences across two points in an individual’s song development and found that the distribution approached that of his tutor’s over time. However, little is known about the extent of vocal sequence learning at various levels of Bengalese finch song organization, about the nature of sequence deviations between tutors and pupils, or about factors that could influence the degree of sequence similarities between tutors and pupils.

## Results

In this study, we examine sequence similarities across the songs of tutors and their pupils at various levels of song organization to assess the nature and extent of vocal sequence learning in Bengalese finches. We divide our analyses into four broad sections. In the first section, we characterize the extent to which the sequence statistics of Bengalese finches raised under semi-natural conditions (“pupils”) reproduced those of their father (“tutor”). In the second section, we present the results of experimental tutoring of juvenile Bengalese finches to bolster the notion that sequence similarities between pupils and their tutors are due to vocal learning. In the third section, we describe in greater detail the nature of sequence similarities and differences between the songs of pupils and tutors and, in the fourth section, we examine how the prevalence, variability, acoustic structure, and timing of elements in the tutor’s song predict the nature and extent of sequence similarities and deviations between pupil and tutor’s songs. Analyses were conducted at multiple levels of song sequencing, including at the level of syllable prevalence (i.e., the probability distribution of syllables in the repertoire), first-order pairwise transitions between all syllables, transitions within stereotyped sequences of syllables (motifs), and transitions at variable sequences of syllables (branch points).

### Sequence similarities between the songs of pupils and tutors

As a first step to understanding the extent of sequence learning in the Bengalese finch, we describe the extent to which Bengalese finches bred under semi-natural conditions reproduce the sequence statistics of their father’s song. For these analyses, we compared the adult song of a breeding male (tutor) to the adult song of his offspring (pupil; see Methods). Pupils’ songs were recorded when they were ~120 days old, at which point their songs are stable and mature^37,42,44,45^.

#### Syllable repertoire

**Figure 2:**
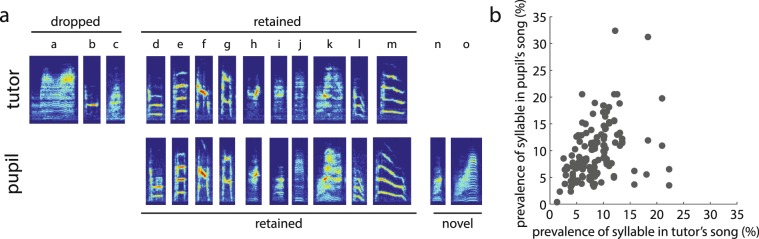
Similarities in syllable repertoires of tutors (fathers) and pupils (sons). (**a**) An example of similarities and differences in the syllable repertoires of a tutor (top) and his pupil (bottom). In this example, 10 of the tutor’s syllables were “retained” in the pupil’s song, whereas three syllables were considered “dropped” by the pupil. The pupil’s song also consisted of two “novel” syllables. (**b**) For retained syllables, the relative prevalence of each syllable in the tutor’s song (as a percentage of the tutor’s repertoire) significantly predicted the relative prevalence of that same syllable in his pupil’s song (n = 157 syllables across 20 tutor-pupil pairs).

A common index of vocal learning is the degree to which the syllable repertoire of the tutor (e.g., father) is reproduced in the repertoire of his pupil (e.g., son). Figure 2a depicts the repertoire of a tutor and his pupil. In this example, 10 of the tutor’s 13 syllable types (“a”, “b”, “c”, etc.; 77%) were produced in the song of the pupil; these syllables were categorized as “retained syllables” (see Methods). Across all tutor-pupil pairs (n = 20 pupils fathered by 9 tutors), on average, 59.0 ± 4.5% (mean ± SEM) of tutor’s syllable types were retained by the pupil (range: 21.4–92.3%). In contrast, only 27.3 ± 3.7% (range: 0–53.8%) of the repertoire of a non-tutor (i.e., an adult male that was not the father of the pupil; see Methods) was considered to be shared with individual pupils, and this degree of sharing was significantly lower than that observed between tutors and pupils (F_1,19_ = 32.0, p < 0.0001). Further, the degree of sharing between non-tutors and pupils was similar to that between tutors and non-tutors (29.9 ± 4.3%).

A subset of a pupil’s repertoire consists of syllable types that were not categorized as tutor syllables (see Methods; “novel syllables”). On average, only 27.3 ± 4.4% of pupil syllable types were categorized as novel (range: 0.0–66.7%), which was significantly less than the percent of syllable types that was considered to be retained from the tutor (F_1,19_ = 27.1, p < 0.0001).

The relative prevalence of a syllable type (i.e., the number of times a syllable type is produced relative to the total number of syllables produced) in a bird’s song can be considered a broad metric of the sequencing statistics of a bird’s song. As such, we calculated the prevalence of each retained syllable relative to all other syllables in each bird’s song for each tutor-pupil pair. (Only retained syllables were analyzed because only these syllables are shared between pupils and tutors). There was an overall significant and positive relationship between the relative prevalences of retained syllables in the repertoires of tutors and of pupils (Fig. 2b; n = 157 syllables; F_1,148.5_ = 26.5, p < 0.0001). As such, these data suggest that pupils generally track and reproduce the prevalence of syllables within their tutor’s song.

#### Pairwise transitions

**Figure 3:**
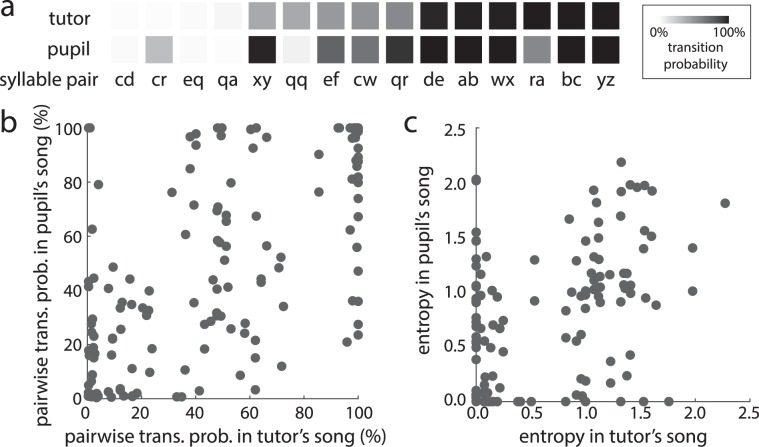
Similarities in syllable pairwise transitions between the songs of tutors (fathers) and pupils (sons). (**a**) An example heatmap of similarities and differences in transition probabilities (from the first syllable of the syllable pair to the second syllable) of all retained pairwise transitions within the tutor-pupil pair from Fig. 2a. (**b**) For retained pairwise transitions, the probability of a transition in a tutor’s song significantly predicted the transition probability of the same pairwise transition in his pupil’s song (n = 168 transitions across 20 tutor-pupil pairs). (**c**) The transition entropy of sequence transitions from retained syllables in the tutor’s song significantly predicted the transition entropy of the same syllable in the pupil’s song.

As a general approach to analyzing sequence learning, we examined similarities in pairwise transition probabilities between retained syllables (“shared transitions”) for each tutor-pupil pair. Overall, pupils retained 43.7 ± 4.1% of their tutor’s transitions following retained syllables (range: 0–77.8%). An example of this comparison is depicted in Fig. 3a. In this example, 12 transitions (out of 24 transitions in the tutor’s song) following retained syllables were shared between the songs of the tutor and pupil. The probability of producing the second syllable after producing the first syllable (i.e., local transition probability) is depicted with a heat map, with black representing a 100% transition probability and white indicating a 0% transition probability. There was a close correspondence between the transition probabilities of shared transitions within the tutor and pupil’s songs in this example, and such a correspondence was relatively consistent across tutor-pupil pairs. As such, transition probabilities of shared transitions were significantly and positively related across all tutors and their pupils (Fig. 3b; n = 172 transitions; F_1,158.8_ = 213.6, p < 0.0001). Prominent features of this relationship were that rare transitions (transition probability < 5%) in the tutor’s song also tended to be rare in the pupil’s song and that highly prevalent transitions (transition probability > 95%) in the tutor’s song also tended to be highly prevalent in the pupil’s song. However, such commonalities were not the only factors driving the significant relationship, since this relationship remained statistically significant even after rare (transition probability < 5%) and highly prevalent transitions (transition probability > 95%) in the tutor and pupil’s songs were excluded from the analysis (F_1,55.1_ = 10.8, p = 0.0018).

To further assess similarities in pairwise transitions between tutors and pupils, we analyzed the degree to which the first order transition entropy (i.e., variability of sequencing; see Methods) following each retained syllable in the tutor’s song could predict the transition entropy in his pupils’ songs. Consistent with the similarities in pairwise transition probabilities, we observed a significant and positive relationship in the entropies of transitions following retained syllables such that syllables with more variable transition probabilities in tutor’s song tended to also have more variable transition probabilities in pupil’s song (Fig. 3c; n = 157 syllables; F_1,150.3_ = 51.0, p < 0.0001).

#### Motifs

While the analysis of global pairwise transitions is a useful first approximation of sequence learning in Bengalese finches, analyzing sequence similarities between the songs of tutors and pupils while taking into consideration the types of sequences that are naturally produced is a more accurate and powerful way to assess sequence learning. Bengalese finch songs consist primarily of a number of different “motifs” strung together in a song bout [see Fig. 1; 3.9 ± 0.3 motifs per tutor (range: 3–6)]. Motifs are relatively stereotyped sequences of syllables (range: 2–7 syllables per motif), and we examined the extent of similarities between the motifs of tutors and pupils.

**Figure 4:**
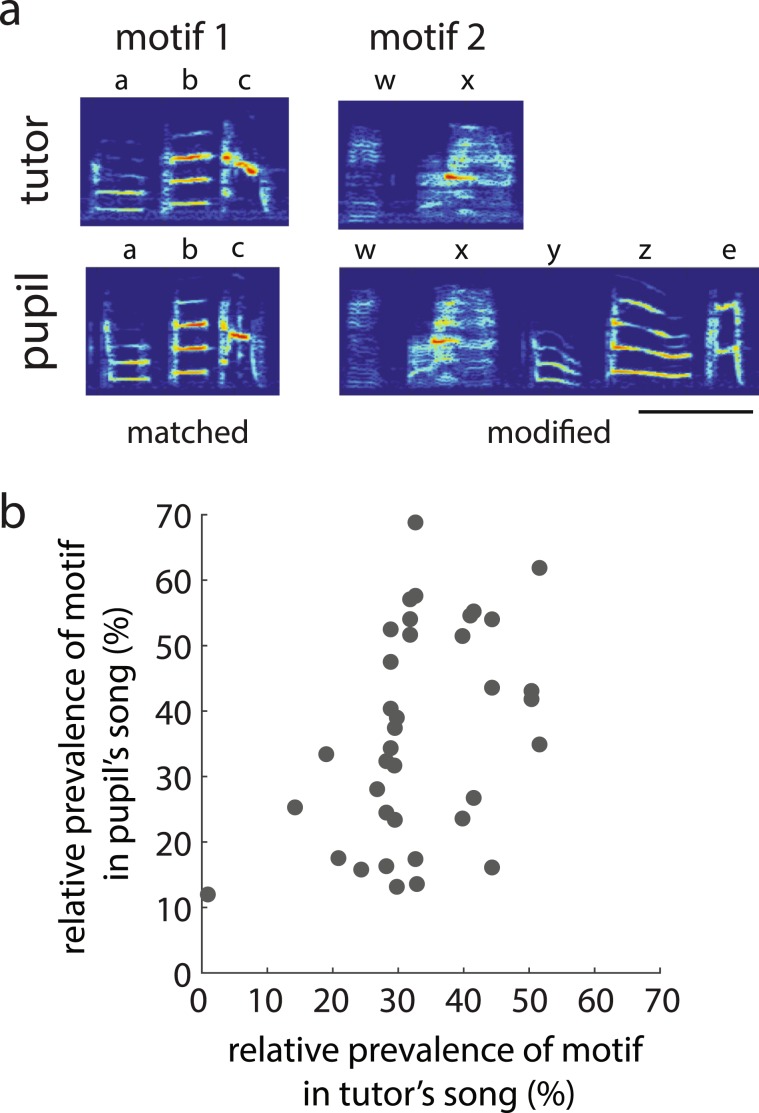
Similarities and differences in motifs between the songs of tutors (fathers) and pupils (sons). (**a**) An example of retained motifs in a tutor-pupil pair. The pupil produced a motif that was classified as “matched” to motif 1 of the tutor’s song (i.e., all syllables and sequences the same) as well a motif that was classified as a “modified” version of motif 2 (i.e., some differences in the syllables in the motif; see Methods). Scale bar = 200 ms. (**b**) The relative prevalence of a tutor’s motif significantly predicted the relative prevalence of the same retained motif in his pupil’s song.

We first independently identified motifs in the songs of tutors and pupils and then quantified the proportion of motifs that were shared between tutors and pupils (see Methods). We categorized a motif as “retained” if ≥50% of the syllables in the motif were shared between the tutor and pupil’s motifs. All 20 pupils retained at least one motif from their tutor’s song. An example of retained motifs in a tutor-pupil pair is depicted in Fig. 4a, and in this example, two of the tutor’s four motifs were retained by the pupil. The pupil produced a “matched” copy of one of the tutor’s motifs (motif 1) and a modified version of the other (motif 2; see Section 3 for a detailed analysis of motif modifications). Overall, 45.0 ± 5.5% of tutor motifs were retained by pupils (range: 16.7–100%).

Just as comparisons of the relative prevalences of retained syllables can be used as broad metrics of sequence similarity, a comparison of the relative prevalences of retained motifs could provide a useful metric of sequence similarity at a “higher” level of sequencing. We observed significant similarities in the relative prevalence of individual retained motifs (i.e., the prevalence of a retained motif relative to all motifs in an individual’s song) across the songs of tutors and pupils (Fig. 4b; n = 37 motifs; F_1,31.1_ = 7.4, p = 0.0106). In other words, motifs that were more commonly produced in a tutor’s song were also more commonly produced in his pupil’s song. This relationship between relative prevalences within the tutor and pupil’s songs did not differ for “matched” and “modified” motifs (interaction between category and prevalence in tutor: F_1,27.3_ = 0.3, p = 0.5644).

#### Branch points

Bengalese finches can produce variable sequences following a motif, and such nodes in song are called branch points. Branch points consist of a branch point sequence (e.g., “abcd” in Fig. 1) followed by a number of types of branch point transitions (e.g., to syllable types “e” and “v” in Fig. 1), with no single type of transition occurring > 95% of the time. We identified, on average, 2.1 ± 0.6 branch points per tutor. Moreover, 17 out of the 20 pupils retained at least one of their tutor’s branch points and, overall, 56% (range: 0–100%) of tutor branch points were considered to be retained by the pupil.

**Figure 5:**
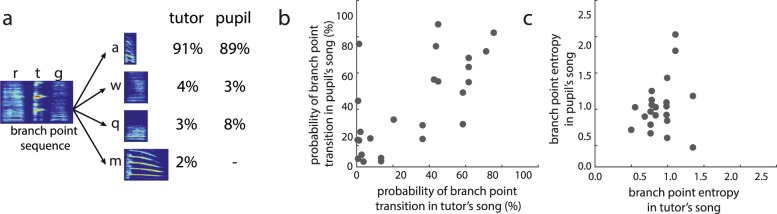
Similarities in syllable sequencing at branch points between the songs of tutors (fathers) and pupils (sons). (**a**) Variable branch points transitions follow branch point sequences. In this example of a retained branch point, the tutor transitioned from the branch point sequence (motif) “rtg” to “a”, “w”, “q” or “m” on 91%, 4%, 3% or 2% of the time, respectively. The pupil retained three of these four branch point transitions and produced them with similar probabilities. (**b**) For retained branch point transitions, the probability of a branch point transition in a tutor’s song significantly predicted the probability of the same branch point transition in his pupils’ songs (n = 26 transitions). (**c**) The entropy of retained branch points in the tutor’s song did not significantly predict the entropy of the branch point in the pupil’s song (n = 22 branch points).

To assess whether tutors and pupils produced branch points with similar sequence probabilities, we compared the probability of downstream syllables following each retained branch point. An example of a retained branch point is depicted in Fig. 5a, where the pupil produced three of the tutor’s four transitions from the branch point sequence (“rtg” as the branch point sequence). Across all retained branch points, pupils produced, on average, 29.6 ± 5.0% of the tutor’s transitions (range: 0–57.5%). The percent of retained transitions at branch points was lower than the percent of retained pairwise transitions (F_1,16_ = 7.1, p = 0.0168). With regard to transition probabilities, within the tutor-pupil pair depicted in Fig. 5a, the tutor and pupil both produced a transition to the “a” syllable, which served as the dominant (highest probability) transition in both the tutor and pupil’s song. Among the dominant transitions that were retained by pupils (i.e., present in tutor and pupil’s songs), 75% of the highest probability transitions in the pupil’s song were also the highest probability transition in the tutor’s song, and the probability of these transitions were similar between the tutor and pupil’s songs (average difference in transition probability: −4.2 ± 6.5%). Furthermore, across all retained branch point transitions, we observed a significant positive correlation such that the probability of the transition in the tutor’s song predicted the probability of that transition in his pupil’s song (Fig. 5b; F_1,21.2_ = 14.0, p = 0.0012).

Despite the relationship between transition probabilities of retained transitions, the entropy of a branch point (variability of sequencing following the branch point sequence) in the tutor’s song did not significantly predict the entropy of the retained branch point in the pupil’s song (Fig. 5c; F_1,18.3_ = 4.7, p = 0.5035). This is likely because there were a number of differences in transitions at retained branch points (see below).

Altogether, these analyses demonstrate that Bengalese finch pupils can track the sequence statistics of their tutor’s song at multiple levels of sequencing and reproduce these statistics in their own songs. Of particular interest is the finding that the probabilities of syllable transitions are significantly correlated between tutors and pupils (Figs. 3b and 5b), as this provides support for a contribution of statistical learning to vocal production learning.

### Analysis of sequence similarity in response to controlled, experimental tutoring

**Figure 6:**
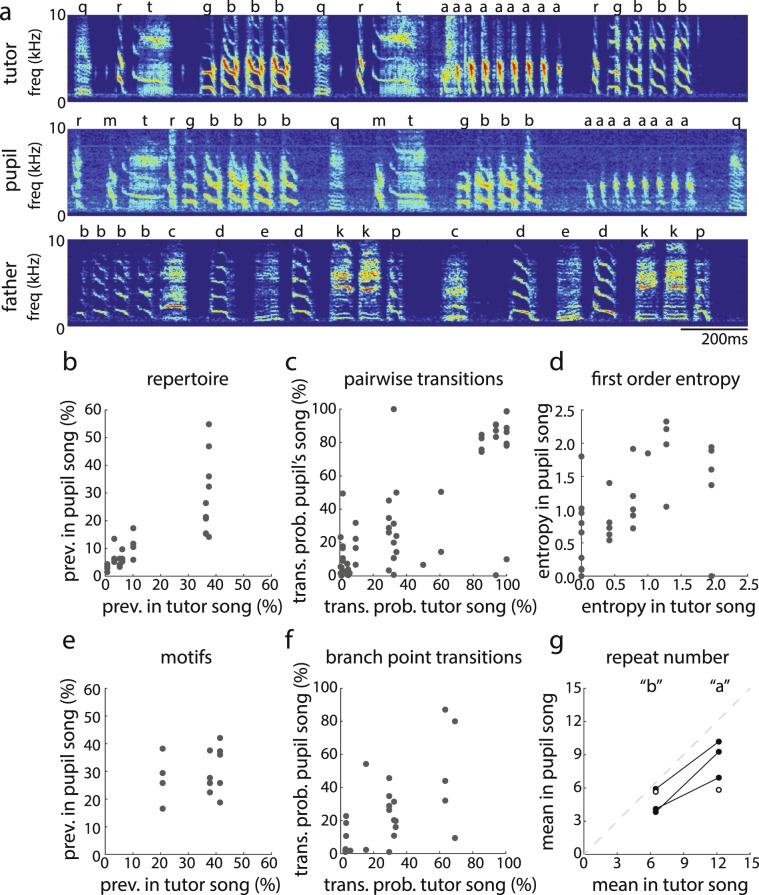
Experimental tutoring confirmed that Bengalese finches learn the sequence structure of their songs. (**a**) Spectrograms of a song in the tutor stimulus (top), a song of the adult pupil (middle), and a song of the pupil’s biological father (bottom). The pupil’s song bears greater resemblance to the tutor stimulus than to his father’s song (see also Supplementary Information). (**b**–**g**) Plotted are song features within the tutor stimulus (x-axis) and the pupil’s song (n = 5; y-axis). The relative prevalence of syllables in the repertoire (**b**), pairwise transition probabilities (**c**), first-order transition entropies (**d**), branch point transition probabilities (**f**), and repeat numbers (**g**) in the tutor stimulus significantly predicted the relative prevalence of these elements in the songs of the pupil. The relative prevalence of motifs in the tutor stimulus, however, did not predict the relative prevalence of motifs in the pupil’s song (**d**). For the data depicted in (**g**), one bird repeated by “a” syllable but not the “b” syllable, and another bird repeated the “b” but not the “a” (white circles).

To confirm that the sequence similarities between tutors and pupils (see Section 1) were not solely due to genetic contributions, we analyzed the extent of sequence learning in response to controlled, experimental tutoring (see Methods). In particular, we passively tutored juvenile Bengalese finches (n = 5 juveniles from three nests) with playbacks of songs of an unfamiliar bird whose song was distinct from the juvenile’s biological father’s song (“tutor stimulus”), and analyzed sequence similarities at multiple levels between the tutor stimulus and the pupils’ adult songs (see example in Fig. 6a).

Similar to the results from the analysis of tutors and pupils raised under semi-natural conditions, there were considerable similarities in syllable repertoires, motifs, and branch points between the tutor stimulus and the songs of pupils. Overall, 75.0 ± 6.9% of syllable types, 93.3 ± 6.7% of motifs, and 53.3 ± 10.0% of transitions at retained branch points in the tutor stimulus were retained in the songs of pupils.

Overall, the relative prevalence of elements and the transition probabilities between syllables in the tutor song stimulus significantly predicted those in the pupils’ songs. For example, the relative prevalence of retained syllables in the syllable repertoire of the tutor song stimulus was significantly related to that in the pupils’ songs (Fig. 6b; F_1,29_ = 42.4, p < 0.0001). A notable feature of this correlation is that two syllables in the tutor stimulus each represented ~35% of the tutor’s repertoire (both are repeated syllables; see below), and both syllables were also quite common in many of the pupils’ repertoires. The relationship remained significant even when these repeated syllables were excluded from the analysis (F_1,23_ = 8.8, p = 0.0080). Pairwise transition probabilities of retained syllables in the tutor song stimulus significantly predicted the transition probabilities of retained syllables in the pupils’ songs (Fig. 6c; F_1,48.7_ = 86.2, p < 0.0001), and the first-order transition entropy following each retained syllable in the tutor stimulus significantly predicted the first-order transition entropy in the pupils’ songs (Fig. 6d; F_1,25,7_ = 12.2, p = 0.0018).

The tutor stimulus contained three motifs. All pupils produced at least one of these motifs in their songs, and pupils produced, on average, 99.3 ± 6.7% (range: 66.7–100%) of the motifs in the tutor stimulus. Despite the imitation of motifs, the relative prevalence of retained motifs in the tutor song stimulus was not significantly related to the relative prevalence of the motifs in the pupils’ songs (Fig. 6e; F_1,9.1_ = 0.4, p = 0.5210).

All three motifs in the tutor stimulus were considered branch points, and all but two (of 14) retained motifs in the pupil’s songs were similarly categorized as branch points. (In these two cases, the transitions from the retained motif were categorized as stereotyped, and the stereotyped transition in the pupil’s song was the dominant transition in the tutor stimulus.) Transition probabilities of retained transitions within retained branch points were significantly correlated between the tutor song stimulus and pupils’ songs (Fig. 6f; F_1,21.9_ = 13.8, p = 0.0012). However, the transition entropy at branch points in the tutor stimulus did not significantly predict the transition entropy following retained branch points in the pupils’ songs (F_1,8.1_ = 0.5, p = 0.5206).

Unlike the tutors’ songs analyzed in Section 1, the tutor stimulus contained motifs that consisted of consecutive repetitions of a single syllable (“repeats”), a common feature of Bengalese finch song^33^. The number of times a syllable is repeated in a motif (“repeat number”) is a characteristic feature of a bird’s song. In the tutor stimulus, both the “a” and “b” syllables were repeated, and repeat numbers for the “a” syllable were ~1.9 times larger than those for the “b” syllable (Fig. 5a). Every pupil retained the “a” and “b” syllables and sequenced at least one of those syllables as a repeat. No other syllable in the pupils’ songs was produced as a repeat. Furthermore, three of the five pupils retained both repeats, and repeat numbers were, on average, 1.9 times larger for the “a” repeat than for the “b” repeat in the pupils’ songs, which is identical to the ratio observed in the tutor stimulus (Fig. 6g). Repeat numbers for both repeats, however, were lower in the pupils’ songs than in the tutor song stimulus.

Importantly, we did not find the same degree of similarities when comparing the experimental pupils’ songs to their biological fathers’ songs (which they were not tutored with; see Methods). (Only four of the five experimental pupils were analyzed here because one of the pupils lacked song recordings of his biological father). Descriptively, one of the pupils did not share any syllables with his biological father; two of the pupils each shared two syllables with their biological father, but these syllables were also present in the tutor stimulus (e.g., syllable “b” in Fig. 6a); and one pupil shared two syllables with both his biological father and tutor, and two syllables with just his father. Overall, pupils shared 22.2 ± 9.1% of their biological father’s syllables, which was significantly lower than the percent of syllables retained from the tutor stimulus (75%; F_1,3_ = 44.2, p = 0.0069). Furthermore, whereas the relative prevalence of retained syllables was correlated between the tutor song stimulus and the pupils’ songs (Fig. 6b), the relative prevalence of syllables shared between a biological father’s song and his offspring’s songs was not significantly correlated (F_1,5.6_ = 0.2, p = 0.6482; Supplementary Figure 1). There were some pairwise transitions that were shared between fathers and pupils (n = 8) but the transition probabilities of these shared transitions were not significantly related (F_1,5.2_ = 0.0, p = 0.9422; Supplementary Figure 1). Similarly, the transition entropies following shared syllables were not correlated between the songs of fathers and pupils (F_1,5.6_ = 1.8, p = 0.2278; Supplementary Figure 1).

Finally, we employed a method to assess similarities between pupils, fathers, and the tutor stimulus that did not involve syllable classification. Specifically, we computed five acoustic features of each rendition of a syllable (see Methods) in the songs of pupils and fathers and in the tutor stimulus, and then compared the distributions of each of these five features between each pupil’s song and the tutor stimulus and between each pupil’s song and its biological father’s song (Supplementary Figure 2). We found that the distribution of acoustic features in a pupil’s song was significantly more similar to the distribution of acoustic features in the tutor stimulus than to the distribution of acoustic features in the songs of their biological father (see Methods; Supplementary Figure 2; Bhattacharyya distance: F_1,35_ = 7.3, p = 0.0107).

Together, these results confirm that juvenile Bengalese finches learn the sequences and acoustic structure of their songs and that sequence similarities between juveniles and their fathers are due primarily to learning and not primarily to genetic similarities.

### The nature of sequence variation between the motifs and branch points of pupils and tutors

The previous sections describe how sequence learning leads to similarities in the sequence structure of the songs of tutors and pupils. However, there are a number of features that vary between the songs of tutors and pupils. In this section, we describe the various ways in which syllable sequencing differed between the songs of tutors and pupils to provide insight into the nature of sequence learning.

#### Pairwise transitions

Whereas sequence transition probabilities and sequence variability following retained syllables covaried between the songs of tutors and pupils, there were a number of differences in sequencing. For example, a number of transitions that were highly prevalent (>95%) in a tutor’s song were less prevalent in the songs of his pupil, and, conversely, a number of transitions that were rare in a tutor’s song were found to be more prevalent in the songs of his pupil. Relatedly, some retained syllables with low or high sequence variability in the tutor’s song were characterized by higher or lower sequence variability, respectively, in the pupil’s songs.

#### Motifs

Overall, 56.3% of the tutors’ motifs were considered to be dropped by their pupils (see Methods for definition of “dropped” motifs). In most cases of dropped motifs (92%), the pupils produced at least one of the syllable types that comprised the dropped motif. However, a majority of syllable types (57%; 96 out of 176) within dropped motifs were dropped from the repertoire of pupils.

As indicated above (Section 1), many of the motifs that were retained in the songs of pupils were modified in some way (23 motifs out of 37 total retained motifs were modified). Motifs were modified by the deletion of one or more syllables (i.e., syllables present in the tutor’s motif but not in the pupil’s motif) and/or the addition of syllables (i.e., syllables produced in the pupil’s motif but not in the tutor’s motif; see Fig. 4a). In most cases, retained syllables within retained motifs appeared in the same order; we only observed one case in which syllables were “shuffled” relative to each other between a tutor and pupil’s motifs [syllables “r” and “t” are sequenced differently in “rtg” (tutor) vs “etr” (pupil)]. Because the fidelity of learning items embedded within a sequence can depend on the position of the item in the sequence^46–49^, we analyzed both additions and deletions according to their motif position (beginning, middle, and end; see Methods).

**Figure 7:**
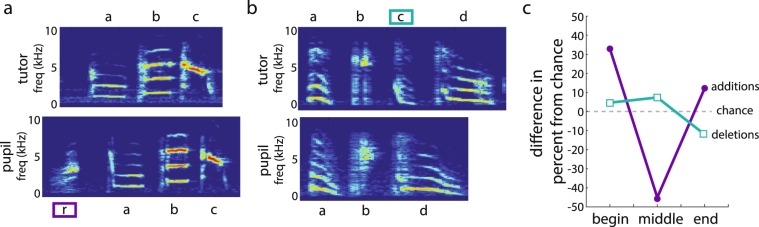
Modifications to motifs. (**a**) An example of a tutor’s motif (top: abc) and his pupil’s motif (bottom: rabc). In this example, the pupil added the “r” syllable to the beginning of the sequence that was the tutor’s motif. (**b**) An example of a tutor’s motif (top: abcd) and his pupil’s motif (bottom), and in this example, the pupil deleted the “c” syllable from the middle of the motif. (**c**) Percent of deletions (teal line and boxes) and additions (purple line and circles) relative to chance (grey line) as a function of position within the motif (i.e., percent of modification observed at a given position minus the percent of modifications expected at that position by chance; see Methods). The distribution for additions was significantly different from chance.

Overall, 44% of motif modifications were deletions and, on average, 1.4 ± 0.2 syllables were deleted in each motif that had been modified with syllable deletions. About half of the syllables (58.3%) that were deleted in modified motifs were syllables that were dropped in the repertoire of the pupil (i.e., produced by the tutor but not by the pupil). With regards to motif position, 33% of deletions occurred at the beginning of the motif, 50% occurred somewhere in the middle of the motif, and 17% occurred at the end of the motif (Fig. 7). To assess the significance of this distribution of syllable deletions, we computed the expected number of syllables to be deleted at each position in the motif given the lengths of motifs in the tutor’s songs (see Methods). Altogether, 24 deletions were observed in total, and the expected numbers of deletions across beginning, middle and end positions were 7, 10, and 7, respectively. The observed occurrences of deletions across these motif positions was 8, 12, and 4, which was not significantly different than the expected distribution (χ^2^_1_ = 1.1, p = 0.5835).

54% of motif modifications were modifications in which pupils added syllables to the tutor’s motif and, on average, 1.5 ± 0.2 syllables were added in each motif with syllable additions. Most (65.5%) syllables that were added to retained motifs were syllables that were retained from the tutor’s song. With regard to motif position, 55% of additions occurred at the beginning of the motif, 10% of additions occurred somewhere in the middle of the motif, and 35% occurred at the end of the motif (Fig. 7). We analyzed whether the observed distribution of additions differed from that expected by chance (see Methods). In total, 29 syllables were added by pupils to the tutors’ motifs and, if additions occurred by chance alone, one would expect 6.5, 16, and 6.5 additions, respectively, at the beginning, middle and end of the motif. The observed distribution of additions was 16, 3, and 10, and this distribution was significantly different from the expected distribution (χ^2^_1_ = 14.7, p = 0.0007). This was driven by the finding that additions were more likely than chance to occur at the beginning of the motif and less likely than chance to occur in the middle of the motif.

In most cases of motif modifications, only a single syllable was added to or deleted from the motif. However, there were five instances in which multiple syllables were added (either to the beginning or end of the motif) and three instances in which multiple syllables were deleted. For the preceding analysis, we counted the number of additions and deletions on a per syllable basis. Using this method, if a pupil deletes the last two syllables from his tutor’s motif, this is scored as an example of deleting one middle and one end syllable. However, if a pupil adds two syllables to the end of his tutor’s motif, this is scored as an example of adding two end syllables. Consequently, this method could differentially inflate estimates of additions at the edge of the motif. Therefore, we also computed and compared expected and observed distributions of additions if instances of multiple additions at a single position were scored as a single addition. Using this method of quantification, we observed additions at the beginning, middle, and end on 12, 3, and 5 instances, respectively. The observed distribution across positions was significantly different from the expected distribution (χ^2^_1_ = 8.4, p = 0.0148) and driven by the facts that additions were more prevalent at the beginning than in the middle of the motif. Therefore, regardless of the number of additions pupils made at each position in the motif, additions were more likely to occur at the beginning of the motif and less likely to occur in the middle of the motif.

Together, the cumulative data on deletions and additions revealed distinct patterns: deletions tended to occur more or less at random within the motif whereas additions tended to occur at the beginning (Fig. 7). Furthermore, both deletions and additions tended to be more prevalent at the beginning of the motif than at the end of the motif.

#### Branch points

Overall, 44% of branch points in the tutor’s song were classified as dropped by the pupil. For most of these instances, the branch point was considered dropped because the motif that served as the branch point sequence was considered dropped from the pupil’s song. However, three motifs that served as a branch point in the tutor’s song were not considered branch points in the pupil’s song because a stereotyped transition (>95%) followed the motif in the pupil’s song. Conversely, two motifs in which transitions were considered stereotyped in the tutor’s song were considered as branch points in the pupil’s song. In four of these five cases where the end of motif was stereotyped in one bird’s song but not the other, the dominant (i.e., most common) transition in the variable bird’s song was the stereotyped transition in the other bird’s song.

Retained branch points were modified in various ways by pupils. While a number of transitions at retained branch points were shared across tutors and pupils, a number of transitions were also dropped. In the example provided in Fig. 5a, the pupil dropped one of the transitions (to the syllable “m”) from the tutor’s branch point sequence (“rtg”). Overall, 69.6 ± 4.9% of transitions from retained branch points were dropped by the pupil, and in most (74%) of these cases, the downstream syllable that was dropped at the branch point was dropped from the repertoire of the pupil (i.e., the pupil did not produce that syllable anywhere in his song).

Retained branch points in a pupil’s song also consisted of transitions that were not produced in the branch point of the tutor’s song (i.e., “novel transitions” within retained branch points). Overall, 60.4 ± 7.1% of all branch point transitions in a pupil’s song were categorized as novel transitions, and in most (77%) of the instances of novel transitions, pupils made transitions to syllables that were not in the tutor’s repertoire (i.e., to “novel syllables”).

### Predicting variation in the extent of sequence similarity between pupils and tutors

In the following section, we discuss factors that could influence the degree of similarities and deviations between the songs of tutors and pupils. In particular, we examined the extent to which the prevalence and sequence stereotypy of elements in the tutor’s song predicted the likelihood of sequence retention or omission and the magnitude of sequence similarity. In addition, we analyzed the degree to which the interval between transitions and distribution of acoustic features could explain variation in the extent of sequence similarity between tutors and pupils.

**Figure 8:**
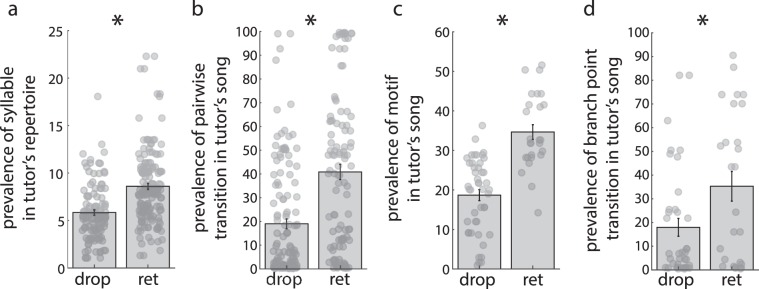
The relative prevalence of elements in the tutor’s song predicted whether elements were dropped or retained by the pupil. (**a**–**d**) Plotted on each panel is the relative prevalence of each element in the tutor song (y-axis) depending on whether that element was dropped or retained by the pupil (x-axis). Each point is an element (e.g., syllable, transition, motif), and bars ± whiskers depict mean ± SEM. Syllables (**a**), pairwise transitions (**b**), motifs (**c**), and branch point transitions (**d**) that were retained by the pupil were more prevalent than elements in the tutor’s song that were dropped by the pupil. *p < 0.05.

We first analyzed whether the prevalence of elements in the tutor’s song was related to the likelihood that pupils would retain or drop an element. For this series of analyses, we considered only cases in which the pupil’s songs contained elements (syllables, motifs, transitions) that were retained as well as elements that were dropped from the tutor’s song (i.e., we excluded cases in which all of a tutor’s elements were retained by the pupil; see Methods). Across all levels of sequencing, elements that were retained by the pupil were more commonly produced in the tutor’s song than elements that were dropped by the pupil (Fig. 8). This was true at the levels of syllable repertoire (Fig. 8a; F_1,261.6_ = 38.8, p < 0.0001), pairwise transitions (Fig. 8b; F_1,251.9_ = 25.6, p < 0.0001), motifs (Fig. 8c; F_1,56.3_ = 58.0, p < 0.0001) and branch point transitions (Fig. 8d; F_1,63_ = 4.8, p = 0.0332). Consequently, across all levels of sequencing, pupils tended to retain song elements that were relatively more prevalent in the tutor’s song, and to drop elements that were relatively less prevalent in their tutor’s song.

However, the prevalence of elements in the tutor’s song did not significantly predict sequence similarity at finer levels. For example, the prevalence of retained motifs in the tutor’s song did not significantly relate to whether the retained motif was categorized as matched or modified by the pupil (F_1,11.0_ = 0.2, p = 0.6460). Similarly, the prevalence of retained branch points in the tutor’s song did not predicted how accurately branch point transition probabilities were copied by the pupil. For this analysis, we computed and then summed the absolute value of all deviations in retained branch point transition probabilities between the songs of tutors and their pupils, and then correlated the sum of these deviations (“total deviations”) for each retained branch point with the relative prevalence of the branch point in the tutor’s song. We did not observe a significant correlation between the prevalence of a branch point in the tutor’s song and the total deviation in transition probabilities (F_1,18.7_ = 0.0, p = 0.8998).

We next assessed the degree to which sequence variability (i.e., entropy) of branch points in the tutor song could influence sequence similarities between tutors and pupils. The entropy of branch points in the tutor’s song was not significantly related to the percent of branch point transitions that were retained from the tutor’s song (F_1,18.4_ = 0.0, p = 0.8551), or to the total deviation in transition probabilities between the tutor and pupil’s branch points (F_1,19.9_ = 0.1, p = 0.7678).

Some studies have reported a link between the interval between successive elements in a tutor’s song and the degree to which that transition is reproduced in a pupil’s song. For example, juvenile white-crowned sparrows that were tutored with single note phrases that were separated by long intervals (e.g., 2.5 sec) did not reproduce the sequences of note phrases^29,50^. As such, their data suggest the possibility that the degree of sequence similarity between tutor and pupil’s songs could be related to the duration of gaps between syllables within the sequence. Phrased differently, it is possible that the sequence statistics of pairwise sequences in the tutor’s songs that are separated by relatively longer gaps could be imitated with a lower degree of accuracy (i.e., similarity) than sequences with relatively short gaps. For this analysis, we assessed the degree to which gap durations at branch points (i.e., the silent interval between the branch point sequence and transition syllable) in the tutor’s song could predict the extent of similarity in the probability of branch point transitions in the pupil’s song. First, we investigated whether the mean gap duration of a branch point transition in the tutor’s song was related to whether that transition was dropped or retained by the pupil. We also examined whether the mean duration of a gap in a retained transition was related to the absolute difference in transition probabilities between the tutor and pupil’s songs. There was no difference in the gap durations of transitions that were dropped vs. retained by the pupil (F_1,52.1_ = 0.01, p = 0.9254), and no significant relationship between gap durations in the tutor’s song and the similarity of transition probabilities between tutor and pupil’s songs (F_1,62.0_ = 2.2, p = 0.1445). These data suggest that gap durations in the tutor do not significantly explain variation in syllable sequencing between the tutor and pupil, though additional experiments are important.

Finally, it is possible that pupils share statistical features with their tutor’s song because of intrinsic biases in the relationship between the acoustic structure of syllables and the sequencing of syllables within Bengalese finch song. If such were the case, tutors could all share some statistical features of their songs. For example, it is possible that the relative prevalence of syllables (Fig. 2b) across the songs of all tutors is correlated with acoustic features of syllables (“prevalence correlation”; e.g., shorter or low frequency syllables could be generally more prevalent than longer or high frequency syllables). To address this possibility, we computed the mean frequency, duration, entropy of the spectral density (“spectral entropy”), entropy of the amplitude envelope (“amplitude entropy”), and spectrotemporal entropy^45,51,52^ of every syllable in songs of tutors. We then computed the median of each feature for each syllable type in the tutor’s song (n = 120 syllables in nine tutors) and then covaried these values with the prevalence of that syllable type (i.e., percent of repertoire). The prevalence of a syllable was not significantly related to any acoustic feature (mixed effects model; p > 0.05 for each feature), indicating the lack of systematic relationships between acoustic features and syllable prevalence. Therefore, the significant correlations between tutor and pupil songs are unlikely to be caused by systematic relationships between acoustic features and syllable prevalence.

## Discussion

Communicative behaviors consist of sequences of elements, and the sequencing of such elements can be learned and can provide various types of information^1,3,53–55^. In contrast to the learning of the acoustic structure of individual elements (e.g., syllables in birdsong), relatively little is known about the acquisition of sequences of acoustic elements, especially for animals with complex sequence structures that resemble those observed in human language (e.g., songbirds like the Bengalese finch). Demonstrating that animals can learn to replicate communicative sequences suggests that they employ statistical learning, an ability to track the relative prevalence of and transition probabilities between elements. Statistical learning is commonly used to describe a learning process that uses the statistical properties of stimuli to segregate or classify communicative elements and has been demonstrated to be important for speech and language acquisition^13,21,31,56–61^. However, vocal sequence learning in songbirds also relies on the ability of the nervous system to extract sequence patterns; consequently, investigations of vocal sequence learning in songbirds can reveal statistical learning processes in non-human animals and provide insight into sensorimotor or cognitive parallels of language acquisition.

By analyzing sequence similarities between the songs of Bengalese finches that serve as tutors and pupils under semi-natural conditions and in response to controlled tutoring, we found evidence that Bengalese finches track sequence patterns at various levels of song organization. Bengalese finches shared much of their syllable repertoire with their tutors and produced songs in which the relative prevalences of and pairwise transition probabilities between learned syllables were similar to those in their tutor’s song. Support for statistical learning also comes from the findings that the transition probabilities of shared branch point transitions were significantly correlated across the songs of tutors and pupils. This latter result in particular suggests that Bengalese finches are not simply forming associations between syllables but also encoding the statistics of syllable transitions.

In addition to revealing the extent and nature of statistical learning in Bengalese finches, we also discovered variables that could predict the degree of sequence similarities between the songs of tutors and pupils. We found that the relative prevalence of syllables or sequences of syllables in the tutor’s song robustly and positively predicted whether a syllable or sequence of syllables was retained or dropped in his pupil’s song. This indicates that syllables and sequences of syllables that are more frequently produced by the tutor are more likely be produced by the pupil. However, we did not observe an effect of relative prevalence on finer levels of syllable sequencing. For example, the relative prevalence of motifs or branch points in a tutor’s song did not significantly predict the extent of sequence similarity between shared motifs or branch points. In summary, the relative prevalence of motifs or branch points in a tutor’s song provided predictive insight into the presence or absence of these types of sequences in the pupil’s song but less insight into the extent of sequence imitation.

In contrast to the relative prevalence of elements in a tutor’s song, the extent of sequence variability (complexity) and syllable timing did not seem to correlate with sequence similarity between the songs of tutors and pupils. For instance, we did not observe systematic differences in the extent of sequence similarities between tutors and pupils at branch points with more or less sequence variability. This lack of an effect of sequence variability on the fidelity of sequence learning is consistent with a previous study in Bengalese finches that did not observe a relationship between the complexity of syllable sequencing and the acquisition of individual syllables^43^. In addition, because syllable sequencing has been shown to be linked to syllable timing^23,40,62,63^, we also examined whether variation in syllable timing could contribute to variation in the extent of sequence learning. However, the duration of the gap of a branch point transition (i.e., the silent interval between the branch point sequence and transition syllable) in the tutor’s song did not predict whether the transition was dropped or retained by the pupil or the extent of similarity in transition probabilities between tutor and pupil’s songs. Given the natural correlations between syllable sequencing and timing in Bengalese finch song^40,62,63^, further experimental approaches are required to reveal the contribution of timing to sequence learning^64^.

In addition to similarities in syllable sequencing between tutors and pupils, our data highlight numerous sequence differences between the songs of tutors and pupils at various levels of song (repertoire, motifs, branch points). On average, ~40% of the tutor’s syllable types (repertoire) were dropped in the song of the pupil, and, conversely, ~30% of the pupil’s repertoire consisted of novel syllables. While we commonly observed pupils retaining many of the syllable transitions from their tutor’s song, some individuals produced very few transitions that were shared with the tutor; for example, one pupil shared 5 syllable types with his tutor but shared only a single pairwise transition with his tutor. Regarding motifs, a little more than half of the tutor’s motif were considered dropped by the pupil (see Methods for definitions) and, among retained motifs, there were a number of instances of additions and deletions of syllables in pupil motifs. In fact, sequencing within a majority (~60%) of retained motifs was modified in some way or another by pupils. Such modifications can lead to the creation of novel syllable transitions as well as the loss of syllables transition, with modifications observed here leading to a small net increase (by 7%) in the diversity of syllable transitions.

Close to half of the branch points in the tutors’ songs were dropped from the pupils’ songs and, within branch points retained in the pupils’ songs, a number of differences in sequence transitions and variability were observed (i.e., dropping of tutor transitions and addition of novel transitions). Among retained branch points, ~70% of transitions in the tutor’s branch point were not observed as transitions within the pupil’s branch point, and the entropies of retained branch points in the tutor and pupil’s songs were not significantly related to each other (despite that the transition entropy of pairwise transitions from retained syllables were correlated between tutor and pupil songs). The extent to which branch points and transitions were dropped in a pupil’s song was related to the prevalence of these elements in his tutor’s song as well as to the extent of repertoire sharing (e.g., many of the dropped transitions at retained branch point were transitions to syllables that were dropped from the pupil’s repertoire).

It is interesting to speculate about the degree to which differences between the songs of tutors and pupils could parallel changes observed across generations for language learning. For instance, there are a number of examples in humans in which variable inputs are “regularized” across generations^65–67^. While we did not observe any systematic reduction in the diversity of syllable transitions or in the prevalence of branch points in the pupil’s song (relative to the tutor’s song), there are some examples that parallel findings in humans. For example, in two instances of dropped branch points, branch points were considered dropped by the pupil because the pupil produced a stereotyped transition after the motif that served as the branch point sequence in the tutor’s song. Interestingly, each of these branch points in the tutor’s song was low in entropy (i.e., low variability in sequencing), and, in both of these instances, it was the dominant transition in the tutor’s branch point that became the stereotyped transition in the pupil’s song. Therefore, mechanisms that underlie the “regularization” of sequences in humans could similarly underlie these phenomena in Bengalese finches.

It has been widely proposed that some deviations between tutors and pupils represent “innovations” on the part of the pupil, and that these innovations serve to distinguish the pupils’ songs from those of their tutors^54^. However, innovation in the context of song learning is often difficult to distinguish from deficits in song learning; both innovation and deficits in song learning (e.g., inability to learn the sensory statistics of the stimuli) manifest themselves as discrepancies between the songs of tutors and pupils. Distinguishing between these hypotheses has important implications for mechanisms of behavioral learning and cultural evolution, especially for the learning of behaviors that may be under selective pressures^68,69^. One example from our study serves as a particularly compelling example of innovation on the part of the pupil. One pupil produced two versions of a single motif of his tutor, with one version being a sequence match to the tutor’s motif and the other being a modified sequence (Supplementary Figure 3). The pupil’s ability to produce a matched version of the tutor’s motif indicates that the pupil accurately encoded the tutor’s song. Simultaneously, the pupil’s production of a modified version of the tutor’s motif suggests a contribution of innovation to song production in pupils. Because such examples provide more compelling support for innovation (and argue against the alternative hypothesis of deficits in learning), it will be important for future studies to identify additional examples of this phenomena.

Our study also highlights that different types of sequence modifications occur in different ways. Motifs can be modified by the addition or deletion of syllables from the tutor’s motifs. Whereas syllable deletions tended to occur relatively randomly within the motif, syllable additions were significantly more likely to occur at the beginning of motifs and less likely to occur in the middle of the motif. The precise mechanisms underlying positional variation in the addition of syllables or variation in the types of motif modifications are unknown, but these findings are generally consistent with the notion that elements occupying different positions within a sequence are differentially encoded and recalled by the nervous system^46–49^.

It is worth noting here that tutors and pupils were different ages when their songs were recorded and analyzed. This is potentially important because syllable sequencing changes over time in adult Bengalese finches^38,39^. For example, sequence variability at branch points decreases over time, due, in part, to a reduction in the number of branch point transitions. However, the vast majority of ways in which the songs of pupils differ from the songs of tutors are not typical of age-dependent changes. For example, whereas differences in syllable repertoire, the presence of motifs, and sequencing within motifs were common between the songs of pupils and tutors, these features of song do not change over time in adult Bengalese finches^38,39^. Therefore, we propose that differences between tutors and pupil are largely related to learning and not age.

In summary, we characterize the nature of statistical learning in the service of vocal production learning, identify some factors that can explain variation in the extent of sequence similarities between tutors and pupils, and provide the first evidence of sequence learning in Bengalese finches under conditions in which the sequence statistics of a tutor’s song is rigorously controlled (i.e., computerized tutoring). Some aspects of vocal sequence learning in Bengalese finches resemble sequence learning phenomena in humans (e.g., regularization, positional variation in phrase modifications), suggesting possible cross-species similarities in mechanisms of sequence learning.

## Methods

### Animals

Bengalese finches (*Lonchura striata* var. *domestica*) were bred at McGill University or Hokkaido University. All birds were housed on a 14 L:10 D or 13 L:11 D light cycle with other birds and provided food and water *ad libitum*. All procedures were conducted in accordance to guidelines and regulations approved by the McGill University Animal Care and Use Committee in accordance with the guidelines of the Canadian Council on Animal Care or by the Committee on Animal Experiments of Hokkaido University based on the national regulations for animal welfare in Japan (Law for the Humane Treatment and Management of Animals; after a partial amendment No. 105, 2011).

### Song collection for analysis of song learning under semi-natural breeding conditions

Bengalese finches from nine nests were bred and raised at McGill University, with each breeding cage visually, but not acoustically, isolated from other birds. Such breeding arrangements are typical for this species and lead to robust song learning of the father’s song^33^. Young Bengalese finches were raised by their father and mother alongside their siblings from the same clutch. Developing birds were removed from their parents’ cage when they were ~60 days old, by which time the sensory period for song learning in Bengalese finches has typically closed^33^, and then housed in same-sex group cages for the rest of development. For this set of analyses, we defined the biological father as the “tutor” for each young Bengalese finch male (i.e., “pupil”). In this species, only males learn and produce song; consequently, only male offspring were compared to their father.

The songs of 20 male offspring were recorded when they were ~4 months old, an age at which song is relatively stable and at which new songs are not learned in normally reared birds^32^. For all song recordings, birds were housed individually in a sound-attenuating chamber (TRA Acoustics, Ontario, Canada), and song was recorded using an omnidirectional microphone (Countryman Associates, Inc, Menlo Park, CA) positioned above the bird’s cage. Computerized, song-activated recording systems were used to detect and digitize song (Sound Analysis Pro: http://ofer.sci.ccny.cuny.edu/html/sound_analysis.html; digitized at 44.1 kHz). Recorded songs were digitally filtered (0.3–10 kHz) for off-line analysis using software custom-written in the Matlab programming language (MathWorks, Natick, MA). All songs recorded and analyzed were spontaneous songs produced in isolation (“undirected song”). The songs of tutors were recorded, on average, 4.8 ± 1.1 months from the period in which pupils were tutored (range: 0.6–9.7 months).

### Song labelling and element identification

For purposes of description and analysis, we use the term “syllable” to refer to individual acoustic elements that are separated from each other by > 5 ms of silence^37–39,44^. Identical to previous studies, we segmented syllables based on amplitude using custom-written scripts in Matlab^34,38,39,63,70^. We tried to analyze >30 song bouts per individual (mean ± SEM: 31.5 ± 1.4; range: 13–47 bouts), with each song being separated by at least 500 ms of silence. Across all birds, 1716 ± 128 (mean ± SEM) syllables were labeled and analyzed per bird, for a total of 51,493 labelled syllables within this set of tutors and pupils.

Because this study centers on sequence similarities between individuals, we underwent a series of steps in the labelling process to ensure that the arbitrary labels for syllable types (“a”, “b”, “c”, etc.) were assigned independent of the syllable’s position within the song sequence. In other words, spectrograms of syllables that were removed from the sequences in which they were embedded were labelled. This labelling process involved two steps: categorization, followed by classification. Specifically, this involved: (1) categorizing syllables produced by each individual bird into syllable “types” independent of sequence (to discern repertoire), and (2) classifying syllables of the same type (i.e., to have the same label) between birds independent of sequence. For the first step (i.e., categorization), one author (L.S.J.) excised a sample of individual syllable spectrograms (i.e., syllable exemplars) from multiple renditions of an individual bird’s song that represented the range of syllables in his song and randomly placed these images of syllables onto a worksheet. The remaining investigators (H.S., K.W., and J.T.S.) individually grouped syllable exemplars into syllable types (e.g., the arbitrary letters above the spectrogram in Fig. 1), without knowledge of the identity of the bird. Syllable exemplars were grouped together based on pairwise “matches” determined by raters in order to determine syllable categorization (i.e., assignment of exemplars into syllable types). Specifically, for each pairwise combination of syllable exemplars on the worksheet, the majority determined whether that pair of exemplars belonged to the same syllable category (i.e., if 2 or 3 of the 3 raters categorized a pair of exemplars together, those exemplars were considered part of the same syllable type). This allowed us to determine the number of distinct syllable types per bird. Estimates of the number of syllables types per bird (syllable repertoire) were highly similar across raters, with each rater differing from the final repertoire size by 1.2 ± 0.1 (mean ± SEM) syllables per bird.

The second step involved comparing syllable types between birds (i.e., classification) so that a common labeling scheme could be used between the birds being compared (e.g., tutors and pupils). For this, the median syllable rendition for each syllable type within a bird was determined by measuring six acoustic features of every syllable rendition (mean frequency, duration, amplitude, spectrotemporal entropy, amplitude entropy and spectral entropy)^45,51,52^ and selecting the syllable rendition that was closest to the centroid (using z-scores for each feature) for that syllable type. A spectrogram of the median syllable rendition for each syllable type (“token”) was placed onto worksheets, with each worksheet containing one token of each syllable type from four birds. Each sheet contained all syllable tokens from at least one tutor and a number of pupils. Some worksheets also contained syllable tokens from a randomly selected “non-tutor” (an adult bird that did not serve as the tutor for the pupils on the sheet). Importantly, raters did not know the identity of the birds on the worksheet. Raters then grouped together tokens that appeared to be of the same syllable type. Each bird was represented with a different colored border around their tokens, and raters were restricted from grouping tokens from the same bird together (since the previous categorization step identified distinct syllable types within each bird). This process allowed raters to not only group syllables from different birds together (e.g., when pupils produced a syllable type that matched his tutor’s syllable type) but also to identify which syllables were not matched among birds on the worksheet. In addition, because some worksheets also contained tokens from non-tutors, this process allowed us to estimate the extent of repertoire sharing between pupils and non-tutors.

The assignment of syllable tokens to shared syllable types was based on majority ruling of groupings, similar to the categorization step. Once again, for each pair of tokens from a tutor and pupil, the majority of raters determined the classification (i.e., if 2 or 3 of the raters grouped a pair of tokens together, they were determined to be the same syllable type). Subsequently, the labels in each pupil and tutor were aligned with the raters’ consensus regarding syllable types and propagated across all the birds’ songs. This classification scheme was used to identify “retained” syllables (syllable types found in both the tutor and pupil’s songs), “novel” syllables (syllable types found in the pupil’s song but not in the tutor’s song), and “dropped” syllables (syllable types found in the tutor’s song but not the pupil’s song). Again, raters were comparable in their classification of syllables; for example, raters differed in their estimates of the number of retained syllable types by 1.5 ± 0.1 syllable per tutor-pupil pair.

To confirm the syllable classifications, we employed the widely-used percent similarity algorithm in Sound Analysis Pro^71^ to compute the acoustic similarity between pairs of syllables that were classified as the same syllable type and between pairs that were not classified as the same. Indeed, syllable pairs of the same type had much higher acoustic similarity than pairs of syllables that were not classified as the same (same type: 77.7 ± 2.5%; not same type: 41.4 ± 2.0%; F_1,19_ = 505.7, p < 0.0001). This supports that our visual approach assigns shared and distinct labels, respectively, to acoustically similar and distinct syllables.

Bouts of Bengalese finch song contain syllables that are arranged in stereotyped sequences (“motifs”) as well as variable sequences of syllables that follow motifs (“branch points”). Song bout durations were 7.6 ± 0.5 (mean ± SEM) seconds and consisted of, on average, 55.5 ± 3.5 syllables. Motifs are sequences of 2–7 syllables that are readily identifiable in Bengalese finch song because of the relatively stereotyped nature of syllable transitions within a motif and because syllables within a motif are generally separated by shorter gaps than the gaps before the first syllable or after the last syllable of the motif^23,62,63^. A branch point is defined as a sequence of syllables that is followed by multiple transitions, with no single syllable transition being produced ≥95% of the time^34,38,39,72^. In our dataset, branch points were motifs in which sequence transitions following the motif were variable from rendition-to-rendition.

Motifs and branch point transitions following branch point sequences (motifs) were identified in the songs of tutors and pupils, with each bird analyzed independently. Within tutor-pupil pairs, the same set of letters was used to label syllables that belonged to the same syllable class in tutors and pupils (as determined from the raters described above). Thereafter, syllables, motifs and branch point transitions within each tutor-pupil pair were categorized as “retained” by the pupil, “dropped” by the pupil, or “novel” in the song of the pupil. Motifs were categorized as “retained” if >50% of syllables in the pupil’s motif were retained syllables in the tutor’s motif.

### Analysis of similarities between the songs of tutors and pupils in semi-natural breeding conditions

We analyzed the similarity between the songs of tutors and their pupils at four complementary “levels” of sequencing: syllable repertoire, pairwise transitions, motifs, and branch point transitions. While analyzing pairwise transitions provides broad insight into sequence structure, analyzing transitions within motifs and branch points is important given the natural organization of Bengalese finch song. One reason for this is that an individual syllable can appear within multiple motifs with stereotyped transitions (e.g., the syllable “c” could appear in two stereotyped motifs - “abcd” and “cefg”), and a global analysis of pairwise transitions would treat transitions from that syllable as variable (e.g., the syllable “c” can transition to “d” or “e”) but the motif-based analysis would treat the transition from that syllable as stereotyped.

For one of our analyses of sequence learning, we analyzed the relative prevalence of elements between the songs of tutors and pupils to provide a broad index of sequence learning. For our analyses of syllable repertoire and motifs, we calculated the prevalence of each element relative to all other elements produced by that bird. In order to calculate the relative prevalence of syllables or motifs in an individual’s song, we divided the number of times each particular syllable or motif, respectively, was produced by the total number of times that element was produced across all of an individual’s songs.

We ran similar, albeit slightly modified, analyses for pairwise transitions and branch point transitions. For these sequencing features, we calculated and compared local transition probabilities. To compute pairwise transition probabilities for each of the bird’s syllables, we counted the number of times a particular syllable transition was produced following a focal syllable type (e.g., “a”, “b”, “c”, etc.), and divided this number by the total number of times the bird produced any transition from the focal syllable type. Typically, Bengalese finches produce 1–4 distinct transitions from any given syllable. To assess similarities in sequence transitions at the level of pairwise transitions, we compared the probabilities of pairwise transitions that were produced by both the tutor and his pupil (i.e., “retained pairwise transitions”, where both syllables in the pair were classified as retained). Similarly, to assess similarities in sequence transitions at the level of branch points, we calculated the probability of every branch point transition following each branch point sequence (see Fig. 5a) and compared transition probabilities of branch point transitions that were produced by a tutor and his pupil (i.e., “retained branch point transitions”). To assess the stability of our estimates of branch point transition probabilities, we randomly split the dataset in half and observed a high correlation for branch point transition probabilities between the halves (r^2^ = 0.9460).

In addition to quantifying similarities between the songs of tutors and pupils, we analyzed sequence deviations at the level of motifs and branch point transitions. To analyze deviations in syllable sequencing within motifs, we identified retained motifs (i.e., ≥50% of syllables in the motif were considered retained) in which the sequence of syllables was not identical between the tutor and pupil (“modified motifs”) due to syllable additions and deletions. Syllable additions were instances in which specific syllables were present in the pupil’s version of the motif but not in the tutor’s. Conversely, syllable deletions were instances in which the syllable was present in the tutor’s version of the motif but not in the pupil’s.

Because positional variation in the fidelity of sequence learning has been observed in other forms of learning^46–49^, we analyzed where within the tutor’s motif these modifications occurred. For this analysis, we separated the tutor’s motif into three sections - “beginning”, “middle”, and “end” - and quantified the number of additions and deletions in each region of the motif. The beginning was defined as the first syllable of the tutor’s motif, the end of the motif was defined as the last syllable of the tutor’s motif, and the middle of the motif consisted of syllables that came after the beginning syllable and before the end syllable of the motif ^52^. As examples of the implementation of these definitions, if the tutor produced the motif “abcd” and the pupil produced the modified motif “rtbcd”, the pupil would be considered to have modified the tutor’s motif by dropping one syllable (“a”) and adding two syllables (“rt”) to the beginning of the motif; if the tutor’s motif was “abcd” and the pupil produced the motif “abcef”, the pupil would be considered to have modified the motif by dropping one syllable (“d”) and adding two syllables (“ef”) to the end of the motif; and, if the tutor produced the motif “abcd” and the pupil produced the motif “abq”, the pupil would be considered to have modified the motif by deleting one syllable (“c”) from the middle of the motif, deleting one syllable (“d”) at the end of the motif, and adding one syllable (“q”) to the end of the motif.

For our analyses of modifications to branch points, we first analyzed the extent to which branch points in a tutor’s song were retained or dropped by his pupil. As noted above, branch points in our datasets were motifs that were followed by variable transitions. Branch points were considered retained if the branch point sequence (a retained motif) was followed by variable transitions in the songs of both the tutor and pupil. For retained branch points, we also quantified the number of branch point transitions that were retained, dropped, or novel, and compared transition probabilities of retained branch point transitions. Branch points in the tutor’s song were considered “dropped” if the tutor’s branch point sequence (motif) was considered to be dropped by the pupil, or if the transition following the tutor’s branch point sequence was stereotyped in the pupil’s song (i.e., a single transition was produced ≥95% of the time following a sequence that was a branch point sequence in the tutor). For these latter instances, pupils and tutors produced comparable sequences; therefore, we could still compare syllable transitions after the sequence between the tutor and pupil’s songs.

Given the extensive documentation of similarities and differences in syllable sequencing between the songs of tutors and pupils (see Results), we aimed to identify factors that could predict the degree of similarity and deviation from the tutor. We hypothesized that the relative prevalence (i.e., abundance) and degree of sequence variability of sequences within the tutor’s song could each affect the similarity of the pupil’s song to the tutor’s song. For our analyses of relative prevalence, we first analyzed the extent to which the relative prevalence of each element (see above for calculation) differed between retained and dropped elements. We ran these analyses at all levels of sequencing (syllable repertoire, pairwise transitions, motifs, and branch point transitions).

The analyses comparing the relative prevalence of dropped and retained elements from the tutor’s song were within-bird comparisons (e.g., to what degree do elements that are retained or dropped by an individual pupil reflect the relative prevalence of these elements in his tutor’s song; see “*Statistical Analyses”* below). Because of this, we excluded cases where all elements were retained, or all elements were dropped by the pupil. For example, if a pupil retained every syllable in his tutor’s repertoire, then we cannot compare the relative prevalence of retained and dropped syllables in his song. Specifically, while no pupils were excluded in our analysis of syllable repertoire sharing (i.e., no pupil retained or dropped every syllable type in the tutor), 2 out of 20 pupils were excluded from the analysis of motifs because they retained all motifs from their tutor (no pupil dropped all of his tutor’s motifs). Additionally, 49 of the 157 shared syllables were excluded from our analysis of pairwise transitions because all transitions from that syllable were retained by the pupil; 33 of the 157 shared syllables were excluded because all transitions from that syllable were dropped by the pupil; and 6 of the retained branch points were excluded because all transitions were dropped (i.e., transitions from the same sequence was variable in both the tutor and pupil but none of the transitions were shared).

We also analyzed how the relative prevalence of motifs and branch points in the tutor’s song co-varied with the fidelity of syllable sequencing. For the analysis of motifs, we analyzed variation in the relative prevalence of tutor motifs that were categorized as matched or modified by the pupils to assess the possibility that tutor motifs that were matched by the pupil were produced more often in the tutor’s song than motifs that were modified by the pupil. For the analysis of retained branch points, we calculated the absolute difference in transition probabilities between the tutor and pupil’s song for every transition produced by either bird. We then summed this difference to calculate the “total deviation in transition probability” for each retained branch point. This value could theoretically range from 0% (pupil perfectly matches the tutor) to 200% (no transition is shared between tutors and pupils); in the example provided in Fig. 5a, the total deviation in transition probability is 10%. We then co-varied this deviation value for each retained branch point with the relative prevalence of the branch point in the tutor’s song.

We analyzed how sequence variability in the tutor’s song could impact the degree of sequence similarities at branch points. For this, we measured the sequence variability of a retained branch point in the tutor’s song and co-varied this measure with the degree of sequence similarity between each tutor and pupil. We computed the *transition entropy* of each branch point in the tutor’s song, which captures the degree of sequence variability^34,38,39,42,70^, using the following formula:

$${\rm{transition}}\,{\rm{entropy}}=\varSigma -{p}_{i}\ast {\log }_{2}({{\rm{p}}}_{{\rm{i}}})$$

where the sum is over all transitions produced at the branch point, and p_i_ is the probability of the i^th^ transition (to a syllable type) across all renditions of the branch point. The transition entropy indicates the number of bits of information required to summarize the extent of variation in syllable transitions. Branch points with transitions that are more variable (i.e., closer to uniform probability among transitions and/or with more distinct types of transitions) have higher transition entropies. Instances in which song was terminated immediately following the branch point were not included in the calculation of entropy. We analyzed how the transition entropy of a tutor’s branch point co-varied with whether the branch point was dropped or retained, with the percent of branch point transitions retained from the tutor, and with the total deviation in transition probabilities between the tutor and pupil’s branch point transitions. We also computed transition entropies within the songs of pupils to compare transition entropies following retained syllables (i.e., first-order transition entropy) and retained branch points between tutors and pupils.

#### Experimental tutoring using passive song playbacks

To confirm that similarities between the songs of fathers (tutors) and sons (pupils) raised under semi-natural breeding conditions were not solely the result of genetic contributions, we experimentally tutored Bengalese finches (n = 5 juveniles from 3 nests; Hokkaido University). Fathers were removed from the nest of young Bengalese finches when juveniles were between 15–25 days old and, when juveniles were nutritionally independent, they were placed individually into a soundbox for song tutoring.

Starting when juveniles were, on average, 30.8 ± 2.7 days old (range: 26–40 days old), pupils were tutored with four song renditions from a single adult Bengalese finch male (“tutor stimulus”) that was not the biological father of any of the juveniles. The tutor stimulus was acoustically distinct from the songs of the fathers of the pupils (e.g., Fig. 6a). Tutor songs were passively played back in a random order, seven times in the morning and seven times in the afternoon. Birds were individually housed and tutored until they were ~120–140 days old, when songs were recorded for analysis.

To reveal the extent of song sequence learning, we compared syllable repertoires, pairwise transitions, motifs and branch point transitions between the tutor stimulus and the adult songs of each pupil (see above for definitions, steps for classification, and calculations). We used identical methods for syllable classification as above, with the addition that each pupil was simultaneously (and blindly) compared to the tutor stimulus as well as his biological father. We analyzed 20.1 ± 2.5 (range: 14–29) songs per bird. Songs were 6.0 ± 0.8 seconds long and consisted of 60.7 ± 7.2 syllables for a total of 8,289 syllables across all recordings.

In addition to a motif like those described earlier, the tutor song stimulus contained two motifs that consisted of repetitions of a single syllable (“repeats”), a common feature of Bengalese finch song. For the analyses of repeats, we compared the average number of times these syllables were consecutively repeated within the motif (“repeat number”) between the songs of tutors and pupils.

We also compared the syllable repertoire of pupils to the syllable repertoire of their biological fathers. Overall, there was very limited sharing of song elements between pupils and their biological fathers, but pupil and father songs could be compared at the levels of the repertoire and pairwise transitions. (No pupil shared a motif with his biological father, so we could not analyze sequence similarities at the level of motifs or the branch point transitions following motifs.)

As further evidence of song learning, we computed five acoustic features (mean frequency, duration, spectrotemporal entropy, amplitude entropy and spectral entropy) of syllables produced within the songs of pupils, tutors, and biological fathers, and compared the distributions of these acoustic features among pupils, tutors, and fathers (Supplementary Figure 2). We then calculated the Bhattacharyya distance, a generalized form of the Mahalanobis distance that measures the difference between distributions, between each of the five distributions of features (one for each feature) in the pupil’s song to the respective feature distributions in the tutor stimulus and in the biological father’s song^73^.

### Statistical analyses

To determine whether sequence statistics of the tutors’ songs significantly predicted the sequence statistics of pupils’ songs, we compared and correlated the relative prevalence of song elements at four levels of sequencing between tutors and pupils (prevalence in repertoire, prevalence of pairwise transition, prevalence of motif and prevalence of branch point transition). For these analyses, we ran mixed-effects models with the prevalence of an element in the tutor’s song (Prevalence-Tutor) as the independent variable and the prevalence of an element in the pupil’s song (Prevalence-Pupil) as the dependent variable. Because each pupil produces songs that contain multiple retained elements, we included pupil ID nested within tutor ID as a random effect. Furthermore, because birds can produce multiple different types of transitions from individual syllable types (e.g., for pairwise transitions) or from individual branch point sequences, we nested syllable ID or branch point ID within pupil ID (again nested within tutor ID) as a random effect in our analyses of sequence similarities at the level of pairwise transitions or branch point transitions. To analyze transition entropies of pairwise transitions and branch point transitions, we ran similar mixed-effects models with Entropy-Tutor as the independent variable, Entropy-Pupil as the dependent variable, and pupil ID nested within tutor ID as a random effect.

An important control for the analysis of song learning between tutors and pupils in semi-natural conditions is to assess the extent of similarities of pupils’ songs to the songs of adult birds outside the tutoring pair. To this end, we quantified the extent of syllable repertoire sharing between a pupil and a non-tutor (i.e., percent of non-tutor’s syllables found in the pupil’s song; see above). Thereafter, we compared the extent of repertoire sharing of each pupil with his tutor to that with a non-tutor, using a mixed effects model with category (tutor vs. non-tutor) as the independent variable and percent of repertoire shared as the dependent variable.

To analyze how the prevalence of elements in the tutor’s song differed between elements that were dropped or retained by the pupil, we ran mixed-effects models with TYPE (dropped vs retained) as the independent variable, and Prevalence-Tutor (prevalence of the element in the tutor’s song) as the dependent variable. Again, because each pupil was represented more than once in the dataset (only data from pupils that produced both retained and dropped elements were included in the analysis), we included pupil ID nested in tutor ID as a random effect. For the analysis of retained vs. dropped pairwise transitions and branch point transitions, we used the same random effects structure as above but also further nested either syllable ID (because birds can have multiple pairwise transitions from a single syllable type or multiple branch point transitions) or branch point ID nested in pupil ID (which was again, nested within tutor ID).

To measure whether the variability of a branch point predicted how accurately the branch point was copied by the pupil, we ran mixed-effects models with the transition entropy of the branch point in the tutor’s song as the independent variable, and the percent of syllables retained or the total percent sequence deviation (see above) as the dependent variable. Because each pupil could be represented more than once in the analysis (multiple shared branch points), we included pupil ID nested within tutor ID as a random effect.

For four analyses, we were unable to reach model convergence with the random effect structure of the model; consequently, we re-ran the model with the random effect as a fixed effect. In these cases, converting the random effect into a fixed effect allowed the model to converge. This was the case for the analysis of (1) the comparison between the percent a pupil’s syllable repertoire that was considered dropped or retained from the tutor (Section 1), (2) the relationship between the repertoire prevalence in the tutor stimulus and in the songs of experimentally tutored birds (Figs. 3 and 6b), (3) the relationship between the prevalence of branch point transitions in the tutor stimulus and in the songs of experimentally tutored birds (Figs. 4 and 6f), and (4) the relationship between the prevalence of branch point transitions in the tutor song and whether the pupil dropped or retained that branch point transition in semi-naturally tutored pupils (Fig. 8d).

We also analyzed the extent to which the distribution of additions or deletions across different motif positions differed from chance. Overall, the Bengalese finch tutors in our colony produced motifs that ranged from 2–7 syllables in length. Based on the lengths of the motifs across tutors, the expected probabilities of additions occurring at the beginning (first syllable of the motif), middle (syllables between the first and last syllable of the motif), or end (last syllable of the motif) were, respectively, 22%, 55% and 22%. Altogether, 29 syllables were added by pupils to the tutors’ motifs and, if additions occurred by chance alone, one would expect 6.5, 16, and 6.5 additions, respectively, at the beginning, middle and end of the motif. The expected probabilities of deletions occurring at the beginning, middle or end were, respectively, 29%, 43% and 29%. Because 24 deletions were observed in total, the expected distribution of deletions across beginning, middle and end positions were 7, 10, and 7, respectively. These expected frequencies of additions and deletions at these positions in the motif were compared to the observed distributions of frequencies of additions and deletions, respectively, using a Likelihood ratio test.

All statistics were done in JMP version 13.2.0, and α = 0.05 for all analyses.

## Supplementary information

Supplementary information

Supplementary information2

Supplementary information3

## Data Availability

The data used for analyses will be made available in supplementary excel files.
