# Supplementary material for: Statistical learning for vocal sequence acquisition in a songbird
Source: Sci Rep. 2020 Feb 10;10:2248. doi: 10.1038/s41598-020-58983-8 (PMC7010765; doi:10.1038/s41598-020-58983-8)
Supplement: Supplementary file 1 — Supplementary information [file 41598_2020_58983_MOESM1_ESM.pdf]

Statistical learning for vocal sequence acquisition in a songbird

Logan S. James, Herie Sun, Kazuhiro Wada, and Jon T. Sakata

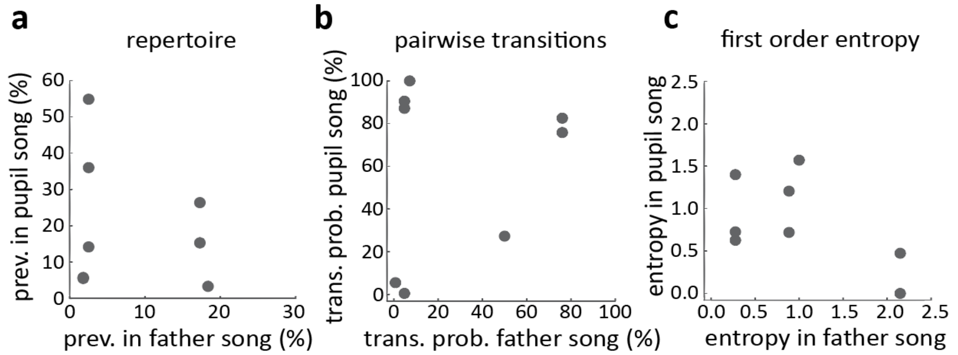

Supplementary Figure 1: Relationship between features of experimentally tutored birds (i.e., tutored by a pre-recorded tutor stimulus; “pupils”; y-axes) and their biological fathers (x-axes). We did not observe significant relationships in the prevalence of syllables that were shared in the repertoires of a pupil and his biological father (a), in the probability of pairwise transitions shared by the pupil and his biological father (b), or the pairwise transition entropy following syllables shared by the pupil and his biological father (c). This lack of correlation with the father’s song contrasts with the numerous correlations with the tutor stimulus (Figure 6).

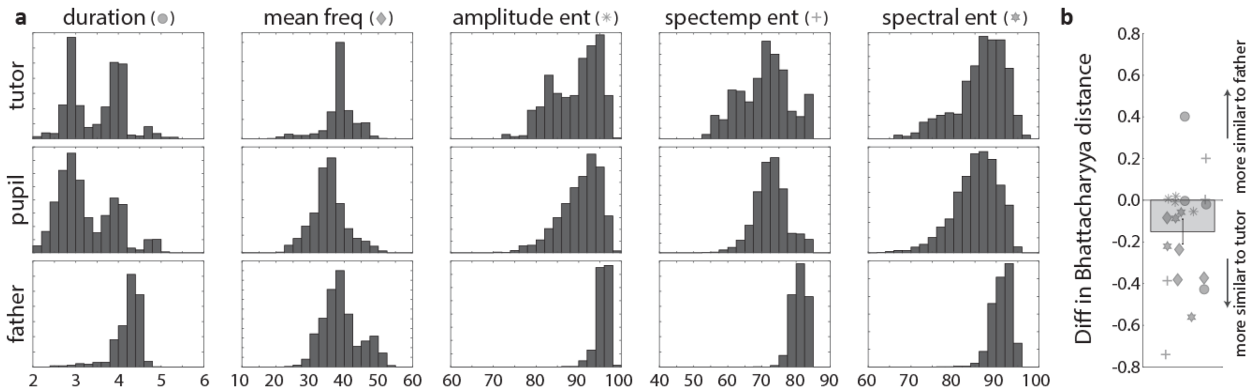

Supplementary Figure 2: Overall acoustic differences between the songs of experimentally tutored pupils, the tutor stimulus, and the pupil's biological father. a. Examples of acoustic feature distributions (histograms) for all syllables analyzed in the tutor stimulus (top), one pupil's song (middle) and his biological father's song (bottom). b. Overall differences between the tutor and pupil distributions compared to the differences between the father and pupil distributions (n=4). Each point depicts the difference between two measures of distance from the pupil distribution, specifically the Bhattacharyya distance between the feature distributions of the pupil and tutor minus the Bhattacharyya distance between the feature distributions of the pupil and father. Negative values indicate that the distribution of the acoustic feature in the pupil's song is more similar to the distribution in the tutor stimulus than the distribution in the biological father. Symbols indicate different features, as depicted in the upper left corner of the tutor histograms in (a).

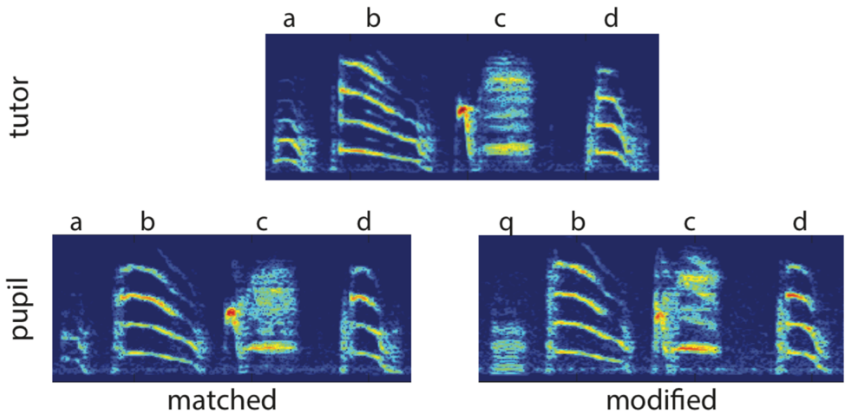

Supplementary Figure 3*: Example spectrograms of a motif in a tutor (top) and two copies of the motif produced by the pupil (bottom). One of the pupil's motifs is a matched copy of the tutor motif (left), whereas his second motif was a modified copy of the tutor's motif ("a" is dropped and "q" is added to the beginning of the motif; right). Such examples provide compelling support that pupils could innovate the sequencing of their tutor's song.*
